# Decreased psychomotor vigilance of female shift workers after working night shifts

**DOI:** 10.1371/journal.pone.0219087

**Published:** 2019-07-05

**Authors:** Thomas Behrens, Katarzyna Burek, Dirk Pallapies, Leoni Kösters, Martin Lehnert, Alexandra Beine, Katharina Wichert, Thomas Kantermann, Céline Vetter, Thomas Brüning, Sylvia Rabstein

**Affiliations:** 1 Institute for Prevention and Occupational Medicine of the German Social Accident Insurance (IPA), Institute of the Ruhr University Bochum, Germany; 2 St. Agnes-Hospital, Bocholt, Klinikum Westmünsterland, Klinik für Unfallchirurgie, Orthopädie und Wirbelsäulenchirurgie, Bocholt, Germany; 3 University of Applied Sciences for Economics and Management (FOM), Essen, Germany; 4 Department of Integrative Physiology, University of Colorado, Boulder, CO, United States of America; University of Wuerzburg, GERMANY

## Abstract

**Background:**

We compared psychomotor vigilance in female shift workers of the Bergmannsheil University Hospital in Bochum, Germany (N = 74, 94% nurses) after day and night shifts.

**Methods:**

Participants performed a 3-minute Psychomotor Vigilance Task (PVT) test bout at the end of two consecutive day and three consecutive night shifts, respectively. Psychomotor vigilance was analyzed with respect to mean reaction time, percentage of lapses and false starts, and throughput as an overall performance score, combining reaction time and error frequencies. We also determined the reaction time coefficient of variation (RTCV) to assess relative reaction time variability after day and night shifts. Further, we examined the influence of shift type (night vs. day) by mixed linear models with associated 95% confidence intervals (CI), adjusted for age, chronotype, study day, season, and the presence of obstructive sleep apnea (OSA).

**Results:**

At the end of a night shift, reaction times were increased (β = 7.64; 95% CI 0.94; 14.35) and the number of lapses higher compared to day shifts (exp(β) = 1.55; 95% CI 1.16–2.08). By contrast, we did not observe differences in the number of false starts between day and night shifts. Throughput was reduced after night shifts (β = -15.52; 95% CI -27.49; -3.46). Reaction times improved across consecutive day and night shifts, whereas the frequency of lapses decreased after the third night. RTCV remained unaffected by both, night shifts and consecutive shift blocks.

**Discussion:**

Our results add to the growing body of literature demonstrating that night-shift work is associated with decreased psychomotor vigilance. As the analysis of RTCV suggests, performance deficits may selectively be driven by few slow reactions at the lower end of the reaction time distribution function. Comparing intra-individual PVT-performances over three consecutive night and two consecutive day shifts, we observed performance improvements after the third night shift. Although a training effect cannot be ruled out, this finding may suggest better adaptation to the night schedule if avoiding fast-changing shift schedules.

## Introduction

Working hours outside 7:00 a.m. to 6:00 p.m. have become common in industrialized societies [1] and may be associated with various negative health outcomes. Next to a presumed association with chronic diseases such as cancer or cardiovascular disease [2,3], shift work involving circadian disruption may disturb the sleep/awake cycle and is frequently associated with poor sleep quality, insomnia, and increased fatigue in shift workers [4].

Increased fatigue during night shifts *inter alia* results from two components: First, there is an increased pressure for sleep with continuing awake time, and, secondly, the pressure to stay awake decreases during the course of the night due to the circadian rhythm of the endogenous circadian clock [5]. Evidence derived from experiments conducted over several days suggests that complex cognitive tasks including task switching are affected by both processes, and that the circadian system may modulate performance across consecutive days of wakefulness [6]. In addition, reaction times may be affected by several sources that can introduce variability in repeated trial performances, including attentional oscillations due to effects of effort variation, subjective state, training, substance intake, and accumulating fatigue [7]. An experimental study suggested that psychomotor vigilance impairment after one single night shift may be greater than impairment observed under blood alcohol concentrations of 0.05%, which is the legal driving limit in many countries [8].

In field studies, however, it is challenging to separate practice effects from other individual sources of variation. Cognitive efficiency is not only influenced by prior duration of rest: Even fatigued subjects may achieve a high degree of reliable performance, especially when simple or automated responses are required. In contrast, more complex tasks, requiring active top-down control of attention, such as monitoring and regulating performance speed and accuracy over extended periods of testing time to stabilize performance control, may be strongly affected by fatigue [9].

To study these processes, psychological experiments have focused on the role of external triggers that may cause an individual to attain peak attention to mobilize efforts on a particular task. On the contrary, an increase in mental focus may result in a higher number of erroneous responses. As the capacity to perform a task is restricted by an overall limit, perception capacity allocation may be channeled to other tasks (i.e. to ensure stability of information throughput to avoid attention failure), which may in addition, be modulated by transient states of low and high motor readiness. Channeling information throughput accordingly, may explain the natural intra-individual variability of performance measures in repeated experimental trials. Indeed, the latter could be of more importance: short-term arousal triggers may not speed up information processing in general, but rather stabilize it against attention failure [10].

Night-shift workers who, due to their work schedule, are forced to change their sleep cycles against their individual needs are particularly affected by psychomotor vigilance impairment. For example, shift workers are at an increased risk for work injuries, but also for drowsiness, car crashes or near-miss automobile incidents while commuting [11–13]. Risks for accidents appear to be most pronounced after the first night shift, followed by a subsequent decrease with additional night shifts. After more than three night shifts per week, accident risk further increases again [13].

Nurses and physicians are particularly vulnerable to develop sleep deprivation due to the need of 24-hour patient care, which requires regular night-working hours [11,14,15]. Several studies demonstrated that nurses and physicians are prone to commit medical and documentation errors while treating patients during night shifts [16,17].

Impaired psychomotor vigilance is a surrogate of fatigue. The Psychomotor Vigilance Task (PVT), which has been validated in several studies, is a sensitive assay to assess neurocognitive effects of sleep deprivation on sustained attention. The test does not require any particular skills and is supposed to be robust against training-related intrapersonal variance, which renders it a simple and suitable tool for the assessment of psychomotor vigilance in epidemiological field studies [18,19]. It should be noted that the PVT is less robust against the impact of transient qualities of subjective states (such as affect, motivation, and cognition), which may counterbalance or enhance other self-regulatory functions, for example those induced by fatigue [20].

The majority of previous field studies among nurses applying the PVT compared psychomotor vigilance between distinct groups of night- and day-shift workers [21–23]. These studies observed decreased response speed or increased lapse frequencies among night- as compared to day-shift workers.

In contrast to these earlier approaches, we applied a within-subject design over the course of several subsequent day and night shifts and taking into account possible confounders, to study differences in psychomotor vigilance after day and night shifts, among female medical staff of a large university hospital. We also examined effect modification by age group, chronotype, and the presence of obstructive sleep apnea on the PVT-performance after each shift block.

## Materials and methods

### Study population and data collection

Between 2012 and 2015, 100 female health care professionals of Bergmannsheil University Hospital staff in Bochum, Germany, working day and irregular night shifts or day shifts exclusively were recruited into the study.

For this analysis we considered only female employees who were working both day and night shifts (n = 75). Eligible participants were 25 years of age or older, not pregnant or breast feeding within the last six months, not taking ovarian stimulation therapy, and did not have a previous diagnosis of cancer. Night shifts (usually three to five per month) lasted from 9 p.m. to 6 a.m. and day shifts from 6 a.m. to 2 p.m. Employees had to have not worked in night shifts at least three days before each study period. Participants were studied during two consecutive day and three consecutive night shifts, respectively. We excluded one woman with severe sleep apnea. Therefore, 74 employees (67 nurses and seven medical lab assistants, hereafter referred to as “nurses” for brevity) were included into the final analysis.

The study protocol was approved by the Ethics Committee of the Faculty of Medicine of the Ruhr University Bochum (No. 4450–12). All participants gave written informed consent.

Participants answered a detailed personal face-to-face interview to assess sociodemographic and lifestyle factors, and anamnestic information at the beginning and end of the study. The Munich Chronotype Questionnaire for Shift Workers (MCTQ^shift^) was used to determine their individual chronotype [24], expressed as midpoint of sleep on work-free days if no alarm clock was used. If a participant had used an alarm clock on work-free days after evening shifts, their mid-sleep on free days after evening shifts was calculated and we used mid-sleep on work days after evening shifts instead. If this was not possible either, mid sleep of free days after morning shifts, and finally mid sleep of work days after morning shifts was used to determine the chronotype. Categorization of chronotype was applied based on the distribution of sleep midpoints among all 100 study participants, using the highest and lowest quartile as cut-off to define late and early chronotypes, respectively. Obstructive sleep apnea (OSA) was determined by the mobile Easy-Screen Pro device (Löwenstein Medical) which records physical activity, pulse, pulse oximetry, systolic blood pressure, respiratory sounds, and air flow. Easy-Screen recordings were evaluated by a sleep expert of the Bergmannsheil Sleep Clinic. A sleep physician diagnosed mild (Respiratory-Disturbance-Index (RDI) 5-≤15), moderate (RDI ≥15-<35), and severe OSA (RDI ≥35) based on episodes of hypopnea and apnea in the Easy-Screen as well as answers in the Epworth Sleepiness Scale questionnaire [25]. Duration of sleep before each shift block was objectively measured by SOMNOwatch^TM^ plus R&K. Because sleep duration was not assessed during the night before the first night shift block, we singly imputed 8-hour sleep duration for the night preceding the first night shift.

### Assessment of psychomotor vigilance

The PVT is a validated instrument to assess neurocognitive effects of sleep deprivation and fatigue-related changes of sustained alertness by repeated reactions to frequent stimuli [18,19]. We applied the brief 3-minute version of the PVT (PVT-B) [26], which is applied as a handheld device, measuring 20cm x 11cm x 5cm and weighing about 660g. Participants had to respond to a visual stimulus (a red diode digital timer) by pressing a response button as quickly as possible, but not too soon, as this would produce a false start. After each reaction, reaction time in milliseconds is briefly displayed. Inter-stimulus intervals vary between one and four seconds. Although a short test may be less sensitive to detect fatigue, the 3-minute PVT-B version was judged to show acceptable sensitivity and specificity for the assessment of fatigue [26].

PVT-measurements were conducted once after a night shift between 5:33 a.m. and 7:44 a.m. when subjects were able to attend the study center located at the hospital. After a day shift, study participants performed a PVT test bout between 1:09 p.m. and 3:41 p.m. Because our study was a real-life field study and was therefore not conducted under standardized conditions, we were not able to control the order of day and night shifts. The study center was a separate office located on the hospital grounds. To keep the test environment fairly standardized and exclude disturbance by other participants, only one nurse was allowed into the room at the same time to perform the test, and there was only limited noise from adjacent rooms. To acquaint nurses with the PVT and to minimize learning effects, all subjects performed a single training session on a work-free day before the first after-shift test performance. Although the PVT may lack test-retest reliability, it can be considered, to date, the most appropriate test to assess cognitive functioning in field studies. Because optimal procedures for computing performance measures are still debated [27], we decided to follow the most commonly recommended practice for assessing psychomotor vigilance in field studies [26]:

1. Reaction time [ms] (excluding lapses and false starts)
2. Reaction time of the slowest and fastest 10% of reactions
3. The percentage of omission errors (“lapses”), defined as reaction times ≥355ms
4. Errors of commission (“false starts”), defined as reaction times prior to or <100ms after the stimulus
5. Throughput as a combined index of response speed and accuracy, calculated as [$\frac{N\;correct\;responses}{Cumulative\;reaction\;times\;(false\;and\;correct)}$] [28].
6. The reaction time coefficient of variation (RTCV) to assess relative reaction time variability [7,10] after day and night shifts, calculated as RTCV = $\left[\frac{SD(reaction\;time\;all\;stimuli)}{mean(reaction\;time\;all\;stimuli)}\right]*100$.

### Statistical analysis

We calculated descriptive characteristics (mean, standard deviation (SD)) of PVT-test performance and other descriptive data for each individual stratified by shift type. We used the paired t-test and the Wilcoxon signed-rank test to test for group differences.

To account for correlated responses between days and shifts for each nurse, we computed linear mixed models with associated 95% confidence intervals (CI). Reaction time and throughput were normally distributed, and therefore an additive model, measuring mean differences, was applied. After log transformation, RTCV showed a normal distribution and was also analyzed applying linear mixed models (multiplicative model). Outcomes related to PVT error percentages were continuous responses between 0 and 1. Assuming that errors in the PVT are governed by a beta distribution, we calculated beta-logistic models (i.e., a multiplicative model, measuring log mean differences in error frequencies). If a participant committed zero errors, we added 0.0000001.

For each model, we included shift type (day shift as reference) as our main independent variable and study day (first day as reference) as fixed effect. In the final models, we included confounders as fixed effects if p was <0.10 in univariate models for one shift type. Models were adjusted for age (per 10 years), chronotype (intermediate as reference), season (winter as reference), and the presence of OSA (yes/no).

To account for each subject’s base response speed, we further adjusted for reaction time in the training PVT in a sensitivity analysis. Another sensitivity analysis included sleep duration before each shift as co-variate.

Analyses were stratified according to age group, chronotype, and presence of OSA. To test for differences between strata, we calculated Cochran’s Q-test for homogeneity in subgroups. For graphic illustration of PVT-outcomes per shift type and study day, we used least squares (LS) means estimates, indicating the model-based average test performance (reaction time or error frequency), adjusted for confounders. For calculation of LS-means, age as linear co-variable was set to the median (34 years).

Statistical analyses were performed with SAS software, version 9.4 (SAS Institute Inc., Cary, NC, USA) and Graph Pad Prism 7.04.

## Results

The study participant’s median age was 34, with an interquartile range from 28 to 47 years. Twenty percent of nurses were classified as early, 49% as intermediate, and 31% as late chronotypes. Further descriptive data are found in Table 1. As it can be expected in such a field study, nurses’ specialties varied widely with the majority working in a general ward or laboratory setting. Twenty-six percent of nurses were employed in an intensive care unit (ICU) (not shown).

**Table 1:** Descriptive data of study population (N = 74 female employees of University Hospital Bergmannsheil, Bochum, working night and day shifts).

Factor	N, median	Percent, Interquartile range
TOTAL [N]	74	100
Chronotype† (median, interquartile range) [hh:mm]	4:08	3:32–5:03
Categories of chronotype† [N, %] Early (<3:11) Intermediate (3:11–4.47) Late (>4:47)	15 36 23	20.3 48.7 31.1
Obstructive sleep apnea [N, %]	20	27.0
Age (median, interquartile range) [years]	34	28–47
Body-mass index (median, interquartile range) [kg/m^2^]	24.8	22.8–29.2
Body-mass index in categories [N %]		
Normal (<25 kg/m^2^)	42	56.8
Overweight (≥25 kg/m^2^)	18	24.3
Obese (≥30 kg/m^2^)	14	18.9
Season, [N, %] Spring Summer Fall Winter	26 19 16 13	35.2 25.7 21.6 17.6

IQR, Interquartile range

† Chronotype assessed as midpoint of sleep by the Munich ChronoType Questionnaire for shift workers (MCTQ^shift^)

Nurses showed higher mean reaction times after a night shift. The mean 10% fastest reaction times were lower after a day shift, as were the slowest 10% of responses. There was no difference in RTCV between day and night shift blocks. Lapse frequency was increased after a night shift. By contrast, we found no statistically significant differences in the number of false starts between shift blocks. Throughput (a combined index of response speed and response accuracy) was decreased after a night shift (Table 2).

**Table 2:** Description of psychomotor vigilance task test outcome measures (female employees of University Hospital Bergmannsheil, Bochum, working night and day shifts).

	Day shift	Night shift	p-value*	
Study days (n)				
1^st^ day	71	71		
2^nd^ day	71	71		
3^rd^ day	-	64		
***Psychomotor vigilance task outcomes***	***Mean (SD)***	***Mean (SD)***		
Mean reaction time [ms]				
Total	234.87 (20.23)	241.74 (23.49)	<0.0001
1^st^ day	235.99 (19.97)	243.81 (23.69)	0.0002	
2^nd^ day	233.74 (20.56)	241.13 (23.15)	<0.0001	
3^rd^ day	n/a	240.13 (23.82)		
Mean slowest 10% reaction time [ms]				
1^st^ day	317.64 (19.99)	322.94 (20.78)	0.009	
2^nd^ day	313.69 (20.68)	318.94 (20.61)	0.009	
3^rd^ day	n/a	319.37(23.28)		
Mean fastest 10% reaction time [ms]				
1^st^ day	184.47 (18.02)	190.98 (21.24)	0.003	
2^nd^ day	183.84 (18.87)	191.06 (19.51)	<0.0001	
3^rd^ day	n/a	186.94 (19.95)5.28 (4.98, 5.86)		
***Psychomotor vigilance task outcomes***	***%***	***%***		
Lapses (reaction time ≥355ms) [%]				
1^st^ day	6.44 (4.62)	9.01 (10.53)	0.0728	
2^nd^ day	6.08 (5.35)	9.19 (9.09)	0.0003	
3^rd^ day	n/a	7.83 (8.04)		
False starts (reaction time <100ms) [%]				
1^st^ day	2.94 (4.81)	3.25 (7.26)	0.3259	
2^nd^ day	2.92 (7.75)	2.91 (5.57)	0.4290	
3^rd^ day	n/a	3.06 (5.21)		
Reaction time coefficient of variation (RTCV)^†^				
1^st^ day	28.70 (9.51)	28.27 (14.19)	0.2056	
2^nd^ day	28.06 (14.80)	28.68 (11.05)	0.7439	
3^rd^ day	n/a	28.66 (11.04)		
Throughput^‡^				
1^st^ day	229.91 (33.25)	216.12 (45.09)	0.0009	
2^nd^ day	233.70 (36.81)	217.30 (46.76)	<0.0001	
3^rd^ day	n/a	223.25 (46.08)		

SD, standard deviation

† RTCV = $\left[\frac{SD(reaction\;time\;all\;stimuli)}{mean(reaction\;time\;all\;stimuli)}\right]*100$

‡ Throughput = [$\frac{N\;correct\;responses}{Cumulative\;reaction\;times\;(false\;and\;correct)}$]

*p-values for differences between day and night shifts from paired t-test for reaction time, RCTV, and throughput and from Wilcoxon signed-rank test for error percentages

The mean response reaction time across shift days was 241,74ms (night shift) vs. 234.87ms (day shift, p<0.0001) (Table 2). In multivariable mixed linear models, working at night had a significant influence on reaction time: at night, each reaction was, on average, 7.6ms slower, as compared to the day shift. Based on the univariate results in Table 2, we calculated the difference in response speed of 0.12 hits per second, corresponding to 21.8 reactions over the 3-minute test duration less as compared to day shifts. Adjustment for baseline speed in the training set did not alter the estimate for the night-work effect (results not shown).

Assessing the relative reaction time variability (RTCV) after day and night shifts in the multivariate model, did not indicate a difference in performance between day vs. night shifts (Table 3).

**Table 3:** Linear mixed effects models and log linear mixed effects models for reaction time [ms], throughput, and RTCV in the PVT in day and night shifts without training test bout, adjusted for various factors and beta-logistic mixed effects model for errors (% of lapses, % of false starts) in the PVT in day and night shifts without training test bout, adjusted for various factors and considering repeated measurements over study days.

Factor	Reference	Category	Model 1*		Model 2**	
*Reaction time*			$\hat{\beta }$	95% CI	$\hat{\beta }$	95% CI
Intercept			217.53	204.15; 230.90	209.06	193.01; 225.12
Shift type	Day shift	Night shift	7.50	0.74; 14.26	7.64	0.94; 14.35
Study day	1^st^ day	2^nd^ day 3^rd^ day	-2.46 -4.03	-4.67; -0.26 -7.08; -0.98	-2.46 -4.06	-4.67; -0.25 -7.11; -1.01
Age (per 10 years)			4.94	1.63; 8.25	6.01	2.32; 9.71
Chronotype	Intermediate	Early Late	-	-	-7.36 1.09	-16.42; 1.71 -6.96; 9.13
Obstructive sleep apnea	No	Yes	-	-	4.55	-2.90; 12.01
Season	Winter	Spring Fall Summer	-	-	1.23 9.79 6.33	-8.20; 10.71 -0.06; 19.65 -3.75; 16.40
***Throughput***						
Intercept			274.65	250.19; 299.12	293.73	265.09; 322.37
Shift type	Day shift	Night shift	-14.84	-27.20; -2.48	-15.52	-27.49; -3.56
Study day	1^st^ day	2^nd^ day 3^rd^ day	2.49 8.68	-1.66; 6.63 2.95; 14.41	2.49 8.71	-1.66; 6.64 2.98; 14.44
Age (per 10 years)			-11.73	-17.78; -5.67	-15.34	-21.93; -8.75
Chronotype	Intermediate	Early Late			19.45 -5.40	3.29; 35.62 -19.74; 8.96
Obstructive sleep apnea	No	Yes			-13.08	-26.38; 0.21
Season	Winter	Spring Fall Summer			2.20 15.12 -3.71	-14.67; 19.06 -32.70; 2.46 -21.68; 14.26
			$exp(\hat{\beta })$	**95% CI**	$exp(\hat{\beta })$	**95% CI**
***RTCV***						
Intercept			21.90	18.20; 26.36	21.05	16.77; 26.41
Shift type	Day shift	Night shift	0.99	0.90; 1.09	0.99	0.90; 1.09
Study day	1^st^ day	2^nd^ day 3^rd^ day	1.00 1.02	0.94; 1.05 0.94; 1.10	1.00 1.02	0.94; 1.05 0.94; 1.10
Age (per 10 years)			1.06	1.01; 1.10	1.07	1.02; 1.13
Chronotype	Intermediate	Early Late			0.91 1.00	0.80; 1.03 0.89; 1.12
OSA	No	Yes			1.01	0.91; 1.12
Season	Winter	Spring Fall Summer			0.99 1.04 0.95	0.86; 1.13 0.91; 1.20 0.82; 1.09
***Lapses [%]***						
Intercept			0.03	0.01; 0.05	0.02	0.01; 0.03
Shift type	Day shift	Night shift	1.49	1.08; 2.06	1.55	1.16; 2.08
Study day	1^st^ day	2^nd^ day 3^rd^ day	0.99 0.83	0.87; 1.12 0.69; 0.98	0.99 0.82	0.87; 1.12 0.69; 0.98
Age (per 10 years)			1.26	1.08; 1.47	1.37	1.18; 1.59
Chronotype	Intermediate	Early Late	-	-	0.63 1.22	0.41; 0.94 0.87; 1.71
Obstructive sleep apnea	No	Yes	-	-	1.50	1.11; 2.03
Season	Winter	Spring Fall Summer	-	-	0.91 1.35 0.95	0.59; 1.40 0.88; 2.06 0.61; 1.51
***False starts [%]***						
Intercept			0.02	0.01; 0.08	0.03	0.01; 0.12
Shift type	Day shift	Night shift	1.05	0.55; 2.02	1.03	0.59; 1.80
Study day	1^st^ day	2^nd^ day 3^rd^ day	0.94 0.95	0.69; 1.29 0.62; 1.46	0.94 0.95	0.71; 1.25 0.65; 1.41
Age (per 10 years)			1.10	0.81; 1.50	1.10	0.82; 1.48
Chronotype	Intermediate	Early Late	-	-	0.71 0.64	0.34; 1.46 0.32; 1.32
Obstructive sleep apnea	No	Yes	-	-	0.64	0.33; 1.27
Season	Winter	Spring Fall Summer	-	-	1.18 0.82 0.63	0.56; 2.45 0.36; 1.86 0.26; 1.51

*Model 1 is adjusted for study day and age.

**Model 2 is adjusted for all variables in the table.

The frequency of lapses at night was increased by 50% (exp(β) = 1.55; 1.16; 2.08), whereas the frequency of false starts was not different during night shifts (exp(β) = 1.03; 95% CI 0.59; 1.80). Subjects with an early chronotype less frequently committed lapses. The presence of OSA was associated with a 50% higher likelihood of lapses (exp(β) = 1.50; 95% CI 1.11–2.03), but not of false starts. Throughput was reduced during night shifts (β = -15.52; 95% CI -27.49; -3.46, Table 3). Adjustment for sleep duration before each shift did not affect the night-work effect.

**Fig 1:**
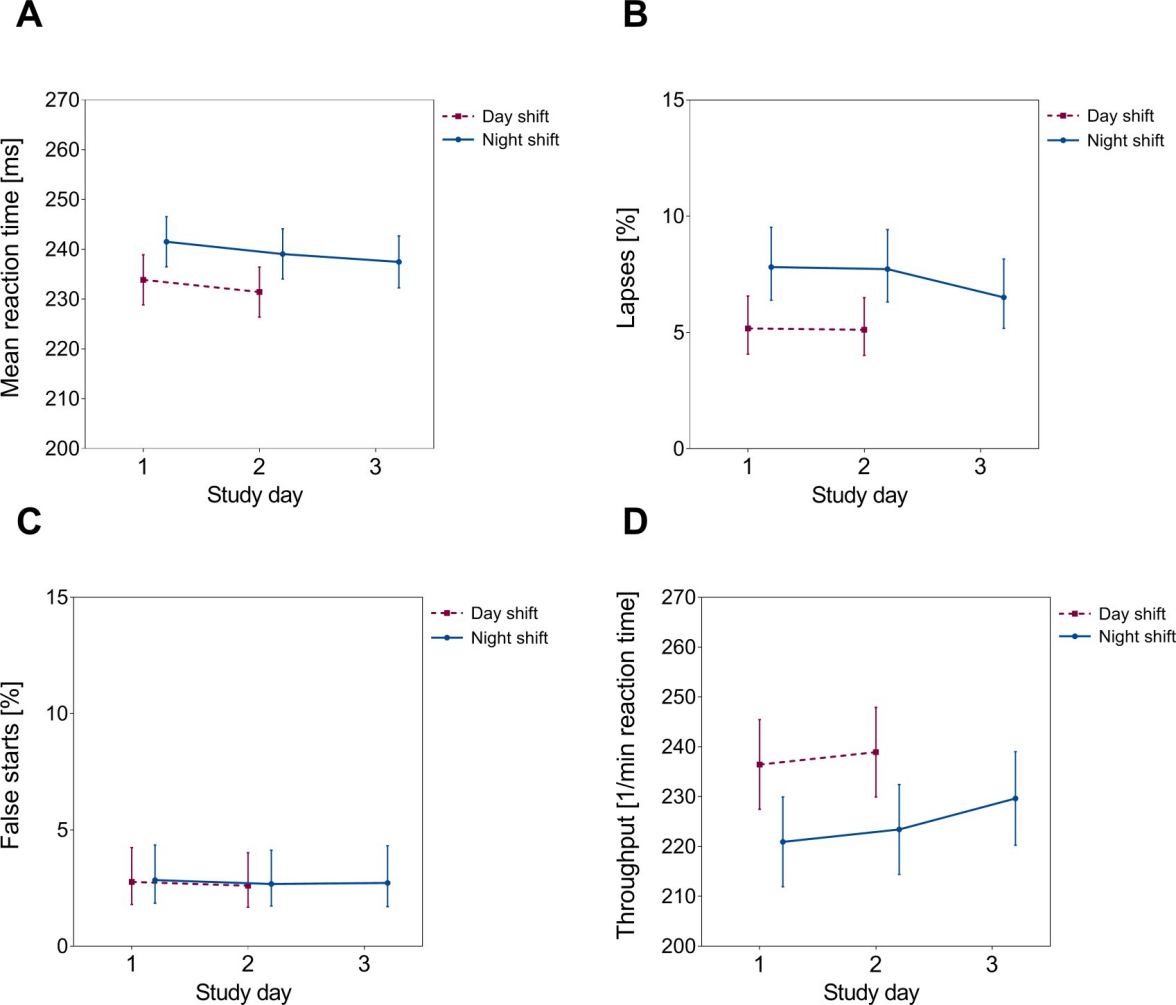
Mean reaction time (panel A) and errors (panels B-D) according to study day: LS-means and associated 95% confidence intervals, adjusted for age, chronotype, OSA and season. Please note that the scale of the y-axis does not start at “0” in panels A and D.

LS-means estimates partitioned by study day, revealed a continuous decrease in estimated reaction times over consecutive shifts after both, day and night shifts. Lapses were, compared to day shifts, approximately 2% higher at the end of the first and the second night shift, respectively. However, the estimated frequency of lapses converged towards the day-shift performance after the third night shift. A similar pattern was seen for the throughput index. In contrast, false starts were not affected by shift types, and did not show a trend across time (Fig 1).

**Fig 2:**
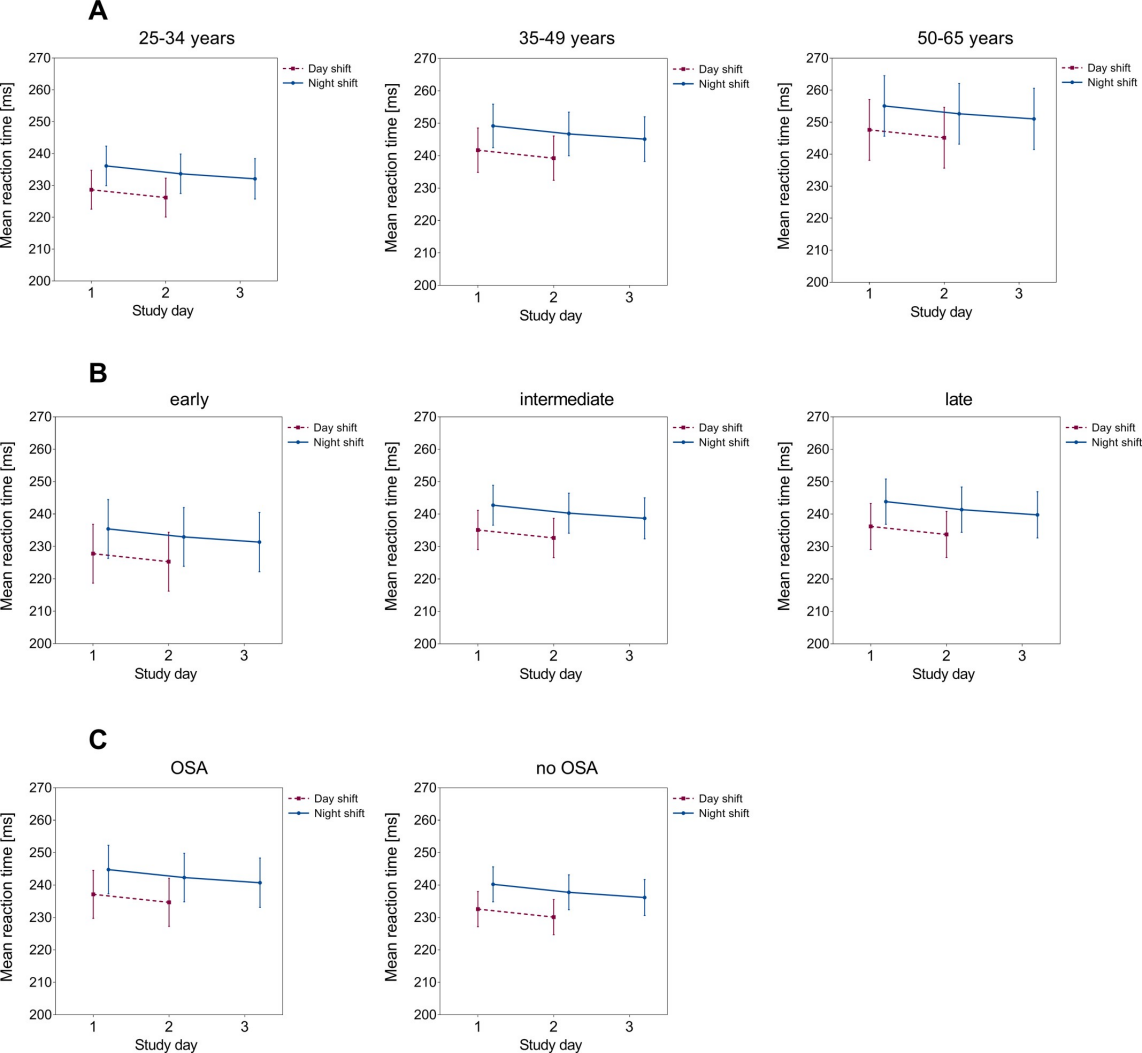
Mean reaction time and associated 95% confidence intervals according to study day: LS-means stratified by age group (panels A, adjusted for chronotype, OSA, season), chronotype (panels B, adjusted for age, OSA, season), and presence of mild to moderate OSA (panels C, adjusted for age, chronotype, season). The scale of the y-axis does not start at “0”.

**Fig 3:**
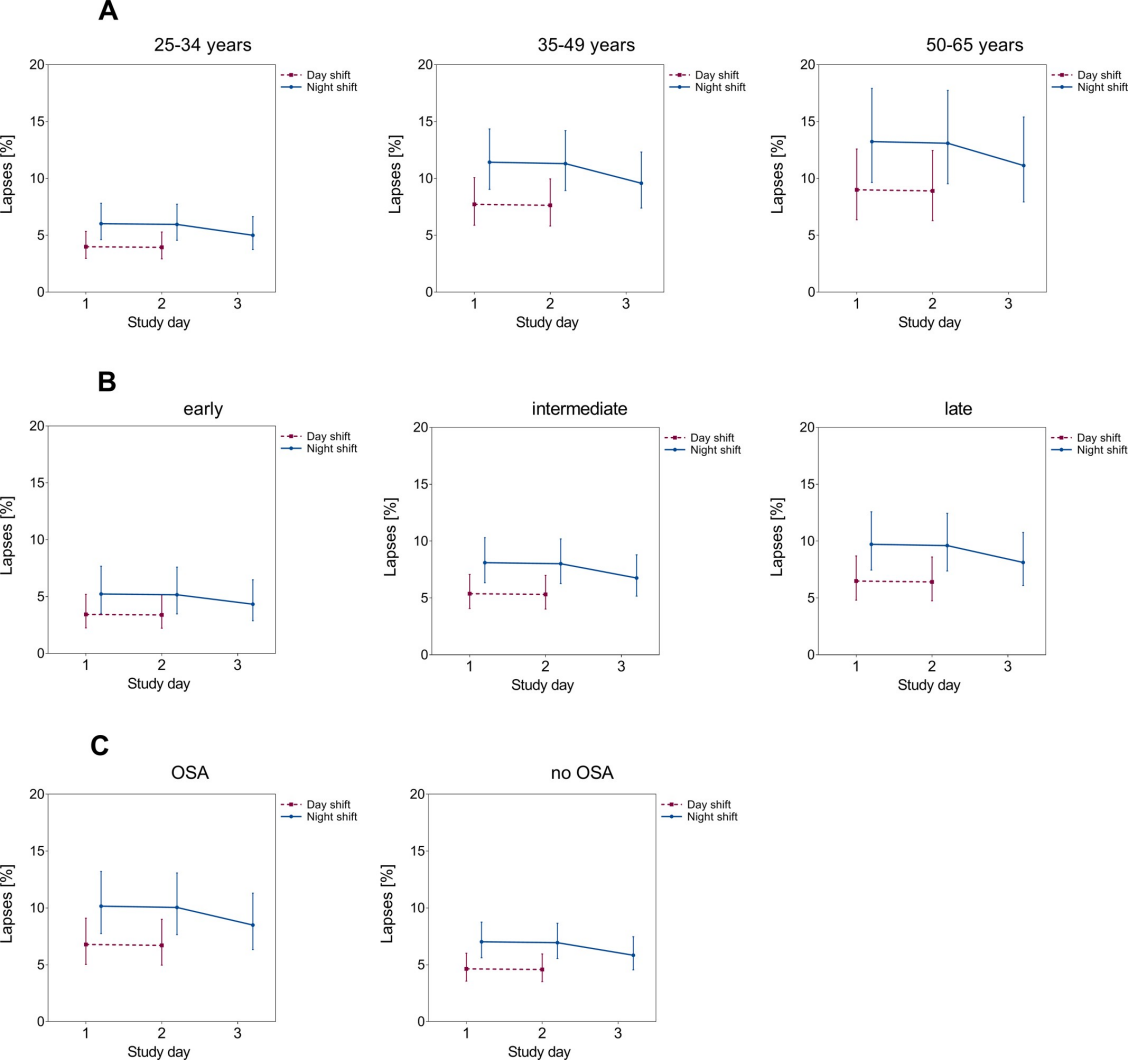
Lapses (%) and associated 95% confidence intervals according to study day: LS-means stratified by age group (panels A, adjusted for chronotype, OSA, season), chronotype (panels B, adjusted for age, OSA, season), and presence of mild to moderate OSA (panels C, adjusted for age, chronotype, season).

The patterns for reaction times and lapses remained similar when partitioning our data according to age group, chronotype, and presence of mild to moderate OSA. Nurses showed decreased psychomotor vigilance with increasing age (Figs 2 and 3, panels A). Likewise, nurses with late and intermediate chronotypes (Figs 2 and 3, panels B) and OSA (Figs 2 and 3, panels C) showed an inferior PVT-performance over the consecutive course of two and three shifts, respectively. However, the observed differences between strata failed to reach the formal level of statistical significance in corresponding stratified analyses (Table B in S1 Tables).

## Discussion

We observed that female hospital workers working rotating shift schedules showed reduced psychomotor vigilance after night shifts as compared to day shifts. Over the consecutive course of three night and two day shifts, we observed an improvement in reaction times in the PVT performance, although RTCV remained unaffected by both, night shift and consecutive shift blocks. The frequency of lapses was reduced after the third night shift, approaching the performance after day shifts. We did not observe differences between day and night shifts for false starts, which remained constant in both groups over subsequent shifts.

Strengths of our field study are the well-defined study population of female shift workers in a university hospital setting, the large sample size compared to similar investigations [21–23], the real-life consecutive monitoring of psychomotor vigilance in the same women over the course of several day and night shifts, and the possibility to control for the influence of numerous confounders.

Limitations include that the study was conducted at a single institution, which hampers generalization of our findings. As comparison shift block, we used early day shifts (starting at 6 a.m.), which still may be the source of relevant circadian disruption [29] that may lead to impairments in psychomotor vigilance, as another within-subject PVT investigation indicated [30]. We did not assess the level of job strain during a shift (i.e. number of co-workers, number of patients or degree and severity of patient care) either, although a study from Finland indicated particularly reduced psychomotor vigilance in nurses during night shifts with high job strain [31]. When we stratified our analysis according to work setting, nurses working in the ICU did not reveal a decreased PVT performance as compared to floor nurses, which is in line with findings of a previous field study [21]. In fact, we only observed a slight tendency for an increased number of false starts, whereas reaction times were decreased and lapses reduced among nurses working the ICU, although differences failed to reach the level of formal statistical significance (not shown). The tendency for a better performance in response speed and lapse frequency observed among ICU nurses may be explained with higher patient alertness required in the ICU setting.

We applied the 3-minute short version of the psychomotor vigilance test (PVT-B) in our field study instead of the standard 10-minute test, which could represent another limitation. We used the PVT-B for practical reasons, and it is obvious that a short test employed to capture sustained attention may be less sensitive to detect fatigue as compared to the longer version. In short tests measurement precision and test reliability usually decrease during the course of the test [32,27]. Indeed, even shorter PVT-versions, a 2-minute and a 90-second version, failed to yield an acceptable sensitivity for the assessment of fatigue due to sleep loss [32,33]. However, the 3-minute PVT-B was judged to show acceptable sensitivity and specificity for the assessment of fatigue in an experimental validation study [26]. However, in field studies one has to trade-off considerations concerning practicality against measurement accuracy.

Further limitations include a rather high variability in PVT performance times after a day and night shift, respectively. However, sensitivity analyses did not indicate an influence of the time of PVT administration on the performance when accounted for in the regression model. The timing of meals during a night shift could also affect PVT performance as a previous laboratory study suggested [34], but due to the real life field character of our study, we were not able to control for this potential source of bias. Likewise, exercise and light exposure, e.g., by walking to the study center, may act as time cues for the circadian system [35], resulting in an alerting effect (similar to a warning signal in experimental psychology, [10]) after a night shift. However, an increase in vigilance after a night shift would rather mitigate the differences in psychomotor vigilance between day and night shifts observed in our study.

The 355ms threshold for an omission error was originally introduced for the PVT-B [26]. Conceptually, the tradeoff between increased performance speed and error frequency, is best captured by interpolated cumulative distributive function analysis (CDF) in chronometric tasks, in which reaction time is also a measure of performance instead of using a fixed threshold for defining lapses [10]. Measures of central tendency indicate that reaction times show a skewed distribution with a steep and narrow left slope due to fast responses and an elongated right tail from a great number of slow responses. Attentional lapses due to involuntary resting pauses, e.g. caused by fatigue, elongate the right tail, but practice effects may counteract this, which aggravates disentangling single effects. As an alternate measure for performance variability closely resembling the analysis of skewness in CDF analysis [10], RTCV has been shown to be less affected by in-between trial variations of response speed and also to be invariant to training effects [7,10]. Assessing RTCV, we did not detect a difference between day and night shifts. Multivariable analysis did not indicate a change of RCTV over consecutive shifts either, indicating that mean reaction time was mainly affected by occasional slow responses at night that, however, do not point toward a general slowing down of information processing.

Our results are in line with the ongoing debate whether omission errors should be considered as true errors or rather very slow reaction times [18]. It is assumed that lapses indeed are an extreme outcome on the continuum of reaction times, but are affected (and continuously increase) by states of sleep deprivation [36]. Extreme PVT-lapses >2,669ms are considered to result truly from microsleep episodes [37]. Our RTCV results may indicate the former, because extreme lapses were extremely rare in our study (N = 3 or 0.12% of all lapses). Likewise, false starts (defined as reaction times prior or <100ms after the stimulus), which may be considered an (over)compensatory mechanism to resist sleep [38], were not increased during night shifts.

An important point is to consider whether short-time activation of attention to increased performance speed may be counterbalanced by an increased error rate as focusing on one task may simultaneously lead to performance deficits (so-called trade-off effects) [10]. This possibility could be suggested by our finding of an improvement in reaction times after subsequent night shifts, but not in error frequencies. We therefore calculated the throughput index, which was designed as a response speed-accuracy combined index that may be dependent on or triggered by external stimuli. Throughput not only appeared to be more sensitive to capture overall cognitive performance, but also has proved to show less variability across several trials compared to reaction times and errors as individual performance parameters [28]. Our results indicate an overall performance decrease across night-shift work, but over consecutive shifts, throughput improved after the third night shift. Interestingly, this improvement rather corresponded with the decrease in lapse frequency observed after the third night (see Fig 1), suggesting a lack of trade-off effects.

Most investigations studying the effects of night-shift work on psychomotor vigilance compared the performance among distinct groups of night- and day-shift workers instead of the intrapersonal variability during different shift blocks [21–23]. However, the results of these studies are largely in line with our findings: A field study from the U.S. comparing psychomotor vigilance among floor and intensive care unit (ICU) nurses in day and night shifts, identified decreased PVT-response speed at night. However, total errors were reduced at the end of a night shift compared to the end of a day shift [21], which is in contrast to our findings. Another field study monitored the psychomotor vigilance of ten female day and six night nurses, working non-rotating shifts [22]. Similar to our findings, day nurses showed slightly faster reaction times after the first and second day of work, whereas response speed before work was comparable between nurses in the two shift types. Wilson and co-workers studying 11 day and 11 night-shift nurses, observed that response speed and the frequency of lapses in the standard 10-minute PVT strongly decreased across night-shift duty time, whereas the performance of day-shift nurses remained unaffected [23]. These results are intriguing as they demonstrate a continuing deterioration of neurocognitive effects due to night-shift work, which is indicative of the two biological mechanisms, increased sleep pressure with continuing time without sleep and reduced pressure to stay awake at night due to the biological circadian rhythm, interacting to induce sleep at night [5,6].

We did not observe a steady decrease in psychomotor vigilance over three consecutive night shifts. Indeed, a previous laboratory study suggested that sleep deprivation accumulated over several night shifts may be rather small and that sufficient sleep is able to mitigate negative effects due to working at night [39]. Controlling for the duration of sleep before each shift, however, did not change our findings of an overall decreased PVT-performance at night.

Across subsequent day and night shifts, reaction times improved, which could be indicative of a training effect. Previous studies suggested a possible learning curve for response speed in the PVT over several test days, which may mask possible effects of sleep deprivation on psychomotor vigilance [39,40], although these effects may be of minor relevance in practice [19]. Indeed, our analysis of RTCV suggested a rather stable performance over time, which seems to imply that practice effects did not influence PVT performance much. However, for lapse frequencies we detected an improvement after the third night shift, when lapses at night converged with the performance during day shifts. This finding may indicate an improved adaptation to the night-shift schedule, which was exactly suggested in an experimental study over a week of simulated consecutive night shifts [8]. A study of psychomotor vigilance among shift-working nurses demonstrated a similar pattern over a 3-day study period: a marginal increase in lapses during the second day and a return to baseline levels on day three [41].

Because improvement was seen in both, day and night shifts, across the first two days, the possible trend towards a recovery of performance after three consecutive night shifts cannot unequivocally be interpreted as evidence for an improved adaptation to the night shift. Because we did not solicit a PVT from participants after the third day shift, it was not possible to evaluate trends in night shifts in comparison with the corresponding trend during the day. This is a general problem associated with studying cognitive performance in field studies in which application of many desirable measures is limited due to practicality considerations.

In summary, we observed reduced psychomotor vigilance among nurses after a night shift compared to their vigilance after a day shift. It cannot be ruled out that these performance deficits were selectively driven by few slow reactions at the lower end of the reaction time distribution function, which may be of less relevance in practice. Although a training effect cannot be ruled out, the observed improvements in lapse frequency suggest that avoidance of irregular or fast-changing shift schedules could lead to a better adaptation to the night schedule. These measures need to be carefully weighed against possible negative effects of a cumulative number of consecutive night shifts, such as increased suppression of melatonin levels and other possible health and social effects, though. Any safeguards taken to promote night health care workers and patient safety requires understanding of the underlying biological processes regarding sleep loss, endogenous circadian rhythms and their effects on complex cognitive tasks.

## Supporting information

S1 Tables (DOCX)
