# Supplementary material for: Decreased psychomotor vigilance of female shift workers after working night shifts
Source: PLoS One. 2019 Jul 5;14(7):e0219087. doi: 10.1371/journal.pone.0219087 (PMC6611661; doi:10.1371/journal.pone.0219087)
Supplement: S1 Tables — (DOCX) [file pone.0219087.s001.docx]

**Table A:** Mixed effects model for slowest and fastest 10% of response speed (ms) in the PVT in day and night shifts without training test bout, adjusted for various factors.

| **Factor** | **Reference** | **Category** | **Model 1***  $\hat{\boldsymbol{\beta}}$ | **95% CI** | **Model 2****  $\hat{\boldsymbol{\beta}}$ | **95% CI** |
| --- | --- | --- | --- | --- | --- | --- |
| ***Mean slowest 10% reaction time*** |  |  |  |  |  |  |
| Intercept |  |  | 304.11 | 291.89; 316.32 | 296.28 | 281.79; 310.78 |
| Shift type | Day shift | Night shift | 5.20 | -0.99; 11.40 | 5.23 | -0.86; 11.31 |
| Study day | 1^st^ day | 2^nd^ day  3^rd^ day | -3.97  -3.32 | -6.89; -1.06  -7.34; 0.70 | -3.97  -3.40 | -6.89; -1.06  -7.42; 0.62 |
| Age (per 10 years) |  |  | 3.60 | 0.60; 6.61 | 4.26 | 0.93; 7.58 |
| Chronotype | Intermediate | Early  Late | - | - | -6.49  -0.43 | -14.64; 1.66  -7.67; 6.81 |
| Obstructive sleep apnea | No | Yes | - | - | 5.32 | -1.39; 12.03 |
| Season | Winter | Spring  Fall  Summer | - | - | 2.84  10.90  7.64 | -5.68; 11.35  2.02; 19.78  -1.43; 16.71 |
| ***Mean fastest 10% response speed*** |  |  |  |  |  |  |
| Intercept |  |  | 163.75 | 152.32; 175.18 | 157.69 | 143.81; 171.56 |
| Shift type | Day shift | Night shift | 6.75 | 0.97; 12.53 | 6.88 | 1.08; 12.68 |
| Study day | 1^st^ day | 2^nd^ day  3^rd^ day | -0.28  -4.84 | -2.46; 1.90  -7.84; -1.83 | -0.28  -4.87 | -2.46; 1.90  -7.88; -1.86 |
| Age (per 10 years) |  |  | 5.46 | 2.64; 8.29 | 6.10 | 2.91; 9.29 |
| Chronotype | Intermediate | Early  Late | - | - | -4.28  1.19 | -12.10; 3.55  -5.75; 8.14 |
| Obstructive sleep apnea | No | Yes | - | - | 3.45 | -2.99; 9.89 |
| Season | Winter | Spring  Fall  Summer | - | - | 0.41  6.76  5.99 | -7.75; 8.58  -1.76; 15.27  -2.72; 14.69 |

*Model 1 is adjusted for study day and age.

**Model 2 is adjusted for all variables in the table.

**Table B:** Linear mixed models for PVT-performance stratified by age, chronotype, and obstructive sleep apnea (OSA)

| **Factor** | **Estimate** | **95% CI** |  | **Estimate** | **95% CI** |  | **Estimate** | **95% CI** | **p-value*^C^*** |
| --- | --- | --- | --- | --- | --- | --- | --- | --- | --- |
|  | **Age group 25-34 years*** | |  | **Age group 35-49 years*** | |  | **Age group 50-65 years*** | |  |
| ***Mean reaction time^A^*** |  |  |  |  |  |  |  |  |  |
| Shift type (night vs day) | 8.41 | -2.29; 19.11 |  | 5.68 | -4.17; 15.52 |  | 13.55 | 4.35; 22.75 | 0.51 |
| ***Lapses*^B^ *[%]*** |  |  |  |  |  |  |  |  |  |
| Shift type (night vs day) | 1.31 | 0.84; 2.04 |  | 1.96 | 1.24; 3.11 |  | 1.47 | 1.02; 2.12 | 0.45 |
| ***False starts*^B^ *[%]*** |  |  |  |  |  |  |  |  |  |
| Shift type (night vs day) | 0.64 | 0.28; 1.48 |  | 1.90 | 0.94; 3.83 |  | 1.18 | 0.48; 2.90 | 0.15 |
| ***Throughput^A^*** |  |  |  |  |  |  |  |  |  |
| Shift type (night vs day) | -11.56 | -29.73; 6.62 |  | -20.06 | -38.73; -1.39 |  | -21.32 | -34.83; -7.81 | 0.69 |
|  |  |  |  |  |  |  |  |  |  |
|  | ***Early chronotype***** | |  | ***Intermediate chronotype***** | |  | ***Late chronotype***** | |  |
| ***Mean reaction time^A^*** |  |  |  |  |  |  |  |  |  |
| Shift type (night vs day) | 3.07 | -10.01; 16.14 |  | 9.23 | -0.35; 18.82 |  | 7.61 | -6.55; 21.77 | 0.76 |
| ***Lapses*^B^ *[%]*** |  |  |  |  |  |  |  |  |  |
| Shift type (night vs day) | 1.25 | 0.76; 2.05 |  | 1.62 | 1.10; 2.37 |  | 1.62 | 0.84; 3.12 | 0.70 |
| ***False starts*^B^ *[%]*** |  |  |  |  |  |  |  |  |  |
| Shift type (night vs day) | 1.22 | 0.55; 2.75 |  | 0.88 | 0.37; 2.08 |  | 1.11 | 0.56; 2.21 | 0.86 |
| ***Throughput^A^*** |  |  |  |  |  |  |  |  |  |
| Shift type (night vs day) | -9.42 | -29.45; 10.61 |  | -18.36 | -35.41; -1.30 |  | -13.20 | -39.48; 13.07 | 0.80 |
|  |  |  |  |  |  |  |  |  |  |
|  | ***Obstructive sleep apnea****** | |  | ***No obstructive sleep apnea****** | |  | | |  |
| ***Mean reaction time^A^*** |  |  |  |  |  |  |  |  |  |
| Shift type (night vs day) | 10.89 | -3.05; 24.84 |  | 6.60 | -1.29; 14.50 |  |  | | 0.60 |
| ***Lapses*^B^ *[%]*** |  |  |  |  |  |  |  |  |  |
| Shift type (night vs day) | 2.18 | 1.12; 4.25 |  | 1.42 | 1.04; 1.94 |  |  |  | 0.25 |
| ***False starts*^B^ *[%]*** |  |  |  |  |  |  |  |  |  |
| Shift type (night vs day) | 0.75 | 0.38; 1.49 |  | 1.12 | 0.56; 2.24 |  |  |  | 0.43 |
| ***Throughput^A^*** |  |  |  |  |  |  |  |  |  |
| Shift type (night vs day) | -23.84 | -51.39; 3.71 |  | -13.50 | -26.72; -0.28 |  |  |  | 0.51 |

*Model is adjusted for study day, chronotype, obstructive sleep apnea, and season

**Model is adjusted for study day, age, obstructive sleep apnea, and season

***Model is adjusted for study day, age, chronotype, and season

*^A^* For mean reaction time, we calculated the additive model, thus $\hat{â}$ is shown.

^B^ For error frequencies and the performance score, a beta-logistic model (multiplicative) was calculated, thus exp($\hat{â})$ is shown.

^C^ Calculated by Cochran’s Q test for homogeneity in subgroups

**Table C:** Sleep duration before each shift block, measured by SOMNOwatch^TM^

| **Sleep duration before shift  [hh:mm]** | **Observations** | **Median** | **Min** | **Max** |
| --- | --- | --- | --- | --- |
| Night shift |  |  |  |  |
| 1^st^ shift | 0 | - | - | - |
| 2^nd^ shift | 66 | 05:24 | 01:18 | 07:55 |
| 3^rd^ shift | 58 | 05:56 | 00:35 | 08:45 |
| Day shift |  |  |  |  |
| 1^st^ shift | 67 | 05:39 | 01:06 | 08:22 |
| 2^nd^ shift | 69 | 05:42 | 00:36 | 07:49 |
